# Polydopamine-based nanospheres as nanoplatforms to kill *Staphylococcus aureus* and to promote wound healing by photothermal therapy

**DOI:** 10.3389/fchem.2022.1111701

**Published:** 2022-12-22

**Authors:** Zhidian Hou, Min Yang, Ling Huang, Songlin Xin, Huiming Yang, Jiangping Hou

**Affiliations:** ^1^ Department of hand and foot surgery, Shandong Provincial Hospital affiliated to Shandong First Medical University, Jinan, Shandong, China; ^2^ Department of Pathology, The First Affiliated Hospital of Shandong First Medical University & Shandong Provincial Qianfoshan Hospital, Jinan, Shandong, China; ^3^ Department of Ophthalmology, Shandong Provincial Hospital affiliated to Shandong First Medical University, Jinan, Shandong, China; ^4^ Department of Otolaryngology-Head and Neck Surgery, Shandong Provincial Hospital affiliated to Shandong First Medical University, Jinan, Shandong, China

**Keywords:** mesoporous polydopamine nanospheres, photothermal therapy, antibacterial, *Staphylococcus aureus*, anti-infective therapy

## Abstract

Bacterial infections have always been a threat when it comes to public health accounting for increased morbidity and mortality rates around the world. For the first time, Polydopamine is often used as an ocular surface drug delivery medium to treat some ocular surface diseases based on its good tissue affinity. Mesoporous polydopamine nanospheres (MPDA NPs) under photothermal therapy (PTT) are demonstrated as efficient therapeutic nanoplatforms for *Staphylococcus aureus* (*S. aureus*) infection and wound healing. MPDA NPs were found to exhibit excellent photothermal performance, significantly causing an increase in temperature within a short period of NIR-I exposure (808 nm, 1 W cm^−2^, 6 min). The MPDA NPs under the NIR irradiation remarkably eliminated *S. aureus in vitro*. Moreover, these synergistic effects turnouts to be phenomenal *in vivo*, effectively killing and healing *S. aureus*-infected abscesses in mice. These revealed the combined effect of the intrinsic antibacterial activity of MPDA NPs enhanced upon NIR-I exposure. Hence, MPDA NPs under NIR-I could prove excellent therapeutic nanoplatforms for bacteria-related infections and other biomedical applications.

## 1 Introduction

In recent years the topic of the ineffectiveness of traditional antibiotics has emerged to be a great concern, although attributed to their misused and over-usage. The other growing concern around the globe concerning this development is the drug resistance of bacteria strains (Wright, 2015; Brown and Wright, 2016; Satpathy et al., 2016). This has amounted to severally associated bacterial infections with others even proving strong tolerance due to their association with biofilms (Blaser, 2016; Zhao et al., 2018; Atkins and He, 2019). A show of a real progressive threat to the public health sector currently and in the years to come, if causative agents are not rapidly dealt with to mitigate their effect. The mention of the chemotherapeutic approach also raises the question of safety and efficacy (Geller et al., 2017; Sougiannis et al., 2019). Thus, the effort to develop alternative means aside from chemotherapeutics, light-activated therapy such as photothermal therapy opens the door of hope and has recently emerged as a promising strategy (Castano et al., 2006; Lucky et al., 2015; Zheng et al., 2016; Li et al., 2023). Under the PTT, bacteria pathogens are rapidly eradicated *via* localized hyperthermia (conversion of laser energy to heat) (Cheng et al., 2014; Abbas et al., 2017; Li et al., 2018). Several nanomaterials could effectively be employed under the PTT, as photothermal agents. However, the single employment of this therapy arouses environmental and public concerns (Xia et al., 2008; Liu and Hurt, 2010; He et al., 2022a). Promotions of versatile nanoplatforms by nanotechnology present the potential of complete combat of this bacteria menace (Liu et al., 2009; Yang et al., 2014; Wang et al., 2015). Hence the construction of biodegradable nanoplatforms with excellent photothermal conversion ability for effective antibacterial therapy remains to be tackled. Polydopamine (PDA) is a kind of biopolymer, a similitude to the natural melanin which has emerged as a photothermal agent. PDA has good adhesion to biological tissues, can effectively adhere to bacteria, *etc.*, and can achieve highly efficient thermal sterilization. PDA has gained attention for its superior NIR absorption property, excellent biocompatibility, stability, mild synthesis process, the presence of functional groups such as catechol and amino groups favorable for modification and excellent biocompatibility essential for *in vivo* experiments, excellent pH sensitivity, *etc.* (Zhang et al., 2015; Farokhi et al., 2019; Wang et al., 2019; Xing et al., 2019; Wang et al., 2021). Mesoporous polydopamine (MPDA) nanoparticles (NPs) have also demonstrated great potential as a therapeutic agent (Huang et al., 2022; Xie et al., 2022), but at present, the photothermal effect, biocompatibility, *in vitro* and *in vivo* antibacterial effect, and the healing of bacterial-infected wound have rarely been reported.

Based on the aforementioned consideration, in this work, MPDA NPs were used to target *Staphylococcus aureus* (*S. aureus*) under NIR-I irradiation. First MPDA NPs were fabricated and characterized. Then their photostability and photothermal efficiency were confirmed. The antibacterial effect *in vitro* and *in vivo* with possible wound healing of *S. aureus* infected abscess, biocompatibility, and their degradation were speculated. Taken together, our study indicated that MPDA may be a potential therapeutic platform for the clinical treatment and rapid healing of *S. aureus*-infected wounds.

## 2 Materials and methods

### 2.1 Materials

1,3,5-trimethylbenzene (TMB), Dopamine hydrochloride, and ethanol was purchased from Aladdin Bio-Chem Technology Co. Ltd. (Shanghai, China) Pluronic F-127, and ammonia solution was purchased from Sigma Aldrich. The working solutions were used in their original state with other preparations made from deionized water.

### 2.2 Synthesis of MPDA NPs

Using the emulsion-induced interface polymerization as reported by (Zhang et al., 2019; Ni et al., 2021; Li et al., 2022). 0.5 g of Pluronic F-127 and 0.8 g of TMB were dissolved in a mixed solvent of water (25 ml) and ethanol (25 ml). 0.6 g of dopamine hydrochloride was added and ultrasonicated until an emulsion solution (milky white) was obtained. Next 2 ml of ammonia was added followed by stirring at 1,000 rpm for 2 h at room temperature. The suspension was further centrifuged at 12,000 rpm for 10 min, precipitates were collected and washed three times with ethanol and water (1:1, *v*/*v*). Finally, ethanol and acetone (2:1, *v*/*v*) were used to remove the emulsion template, precipitates were dried to give MPDA NPs. The black powder MPDA NPs is obtained by vacuum drying.

### 2.3 Characterization of MPDA NPs

The morphology, pore characters, and elemental mapping distributions were observed with transmission electron microscopy (TEM). Talos F200S, Thermo Fisher Scientific (United Stated) operated under 200 kV *via* dropping of relevant solutions on a carbon-coated copper grid.

### 2.4 NIR-induced photothermal performance of MPDA NPs

The photothermal performance and efficiency of MPDA NPs suspension of series of concentration (0–300 μg ml^−1^) were investigated with an 808 nm-laser light source of power density 1 W cm^−2^ for 6 min. Photothermal stability was investigated by five heating and cooling cycles. An infrared thermal imager was used to monitor, record, and take pictures of the temperature changes within a determined time point. The photothermal conversion efficiency was calculated according to previous reports (Roper and Ahnand W Hoepfner, 2007; Younis et al., 2019).

### 2.5 *In Vitro* antibacterial assay

The *in vitro* antibacterial effect of MDPA NPs against *S. aureus* was evaluated. 50 µL of *S. aureus* suspension (O.D_600_ = 0.05) was incubated with 200 μg ml^−1^ of MDPA NPs suspension at 37°C, for 30 min. After the coincubation, the mixture was exposed to laser irradiation (808 nm, 1 W cm^−2^) for 6 min. Finally, the treated bacteria suspension was serially diluted and 100 µL plated on a sterile LB plate. Samples were incubated at 37°C for 12 h. The quantification of the bacterial cells was done by counting colonies (CFU ml^−1^) to determine the survival rate.

### 2.6 *In Vivo* antibacterial assay

Evaluation of the *in vivo* antibacterial effect of MPDA NPs was done by creating a subcutaneous abscess on the BALB/c mice (7 weeks) by first shaving and disinfecting the area with (75% ethanol) immediately after anesthesia 50 µL (1 × 10^8^ CFU ml^−1^) was subcutaneously injected on the back to create abscess which was visible within 48 h. Mice were divided into two groups: PBS (control group) MPDA NPs + NIR (treatment group), with each group containing five mice. PBS and MPDA NPs suspension were later injected into the abscess. The treatment group was exposed to irradiation (808 nm, 1 W cm^−2^, 6 min). An infrared thermal imager was used to monitor and record the temperature changes within the specified time. The infected tissues were isolated, crushed, and cultured in the medium, and the bacteria were counted by the plate method. Animal experiments were performed according to the protocols approved by the Animal Ethics Committee of Provincial Hospital Affiliated to Shandong First Medical University.

### 2.7 Toxicological analysis of tissue

The abscess progress and wound healing was monitored for 14 days. Mice were sacrificed at the end of the 14th day. The skin tissues and the major organs harvested were fixed with paraformaldehyde solution (4%) for hematoxylin and eosin (H&E), Gram, Masson staining and immunofluorescence analysis, TGF-β/CD206 and TNF-α/CD86 primary antibodies with their corresponding antibodies were employed in the process. Mice were carefully discarded in strict accordance with the protocol. Immunofluorescence images were obtained from fluorescence microscopy (Olympus, Japan).

### 2.8 Statical analysis

Data were expressed as mean ± standard deviation, and analysis among groups was determined for statistical significance with a standard Student t-test using the graph pad prism 9.0. Data are presented as mean ± SD (*n* = 3), ***p < 0.001.

## 3 Result and discussion

### 3.1 Preparation and characterization of MPDA NPs

**FIGURE 1:**
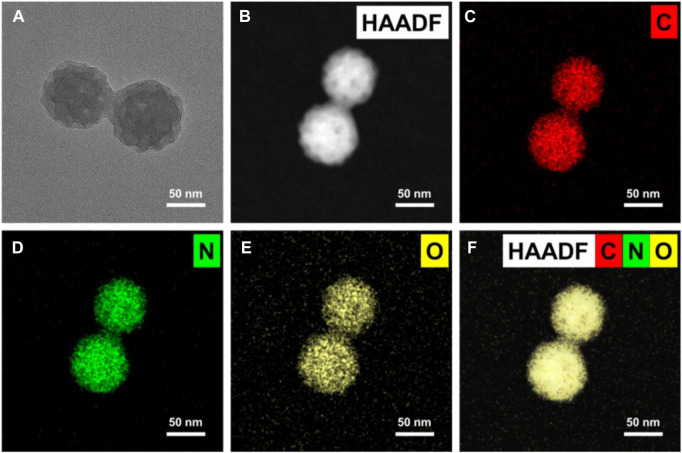
Microscopy characterizations of mesoporous polydopamine particles (MPDA). **(A)** TEM image showing an individual particle with radially oriented mesochannels. **(B–F)** Corresponding EDX mapping of C, N, and O element signals of MPDA.

MPDA was synthesized *via* emulsion-induced interface polymerization as outlined in the experimental section. As shown in Figure 1A, images of transmission electron microscopy (TEM) displayed homogenous spheres with uniform distribution of particle size of the mesoporous structure with pore sizes of approximately 15–17 nm. Next, the images of elemental mapping illustrated that MPDA was made up of carbon, nitrogen, and oxygen, Figures 1B–F, an indication of the perfect distribution of elements within the MPDA and the maintenance of its structure.

### 3.2 Photothermal property of MPDA

**FIGURE 2:**
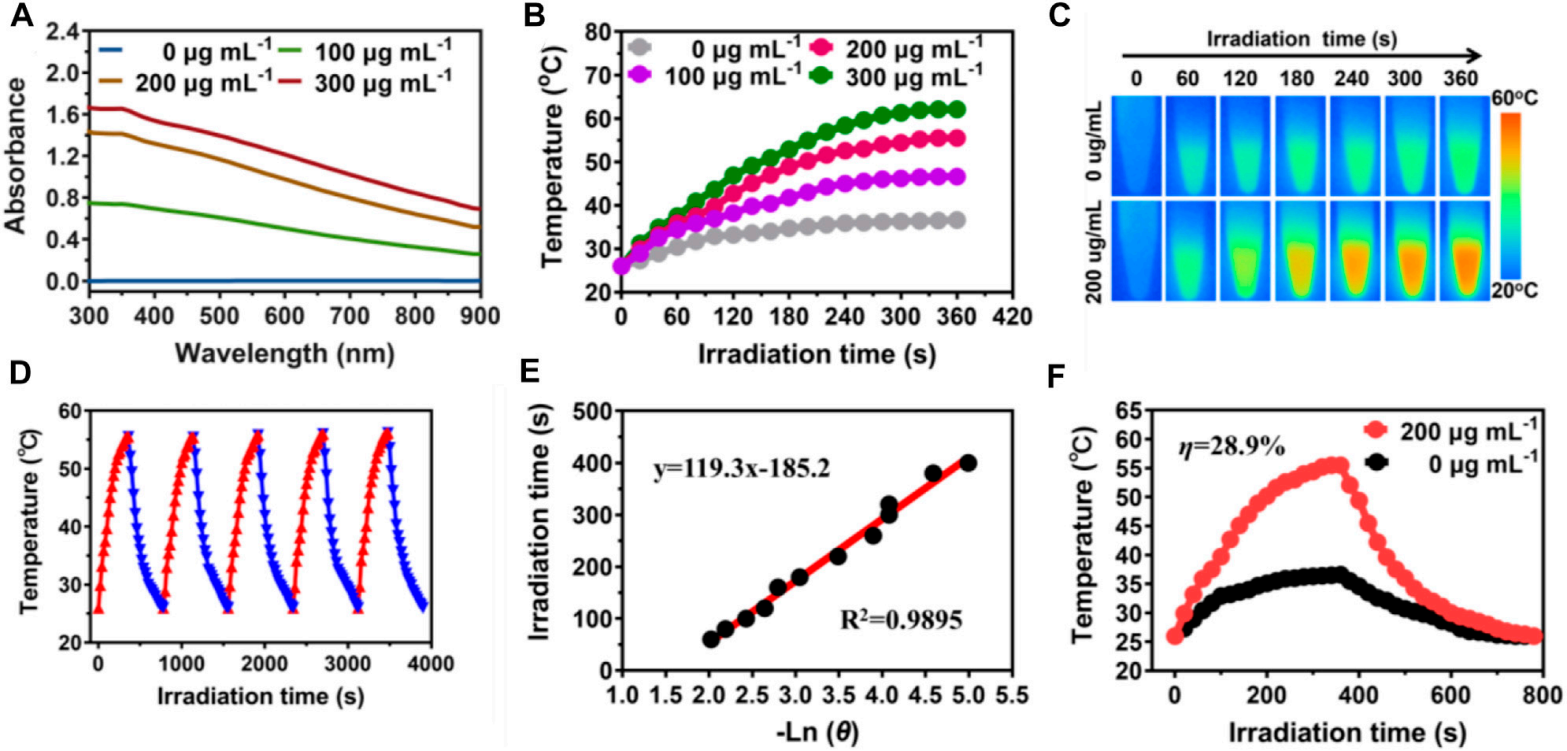
Photothermal properties of MPDA. **(A)** UV-vis-NIR absorption spectra of MPDA dispersions at different concentrations. **(B)** Photothermal heating curves of MPDA dispersions at different concentrations under 808 nm laser irradiation (1 W cm^−2^). **(C)** Infrared thermal images of water and MPDA (200 μg ml^−1^) under irradiation (808 nm, 1 W cm^−2^). **(D)** Photothermal stability (five ON/OFF laser cycles) of MPDA dispersion (200 μg ml^−1^). **(E)** Linear fitting plots of time *versus* ln *θ* during the cooling period. **(F)** The photothermal effect of MPDA dispersions (200 μg/ml, red line) under irradiation (808 nm, 1 W cm^−2^, 6 min) and then switched off the laser (black line is pure water as the control).

Excellent photothermal absorption and conversion efficiency are key when it comes to photothermal agents (Wang et al., 2017; He et al., 2022b). The UV-vis-NIR absorbance of MPDA NPs increased linearly upon concentration increase demonstrating MPDA NPs could absorb the NIR-I light energy, Figure 2A. To evaluate the photothermal effect, a series of concentrations of MPDA NPs suspension (0–300 μg ml^−1^) were irradiated with a NIR-I laser (808 nm, 1 W cm^−2^) for 6 min Figure 2B. Upon irradiation, MPDA NPs suspension exhibited a remarkable linear temperature rise, particularly when the concentration was 300 μg ml^−1^, the temperature rise was 65°C, Figures 2B, C. The photothermal effect was concentration and time-dependent. As shown in Figure 2D, MPDA NPs temperature under five heating and cooling cycles displayed no significant attenuation which indicated good photothermal stability. The photothermal conversion efficiency was determined to be 28.9% Figures 2E, F, which surpassed that of traditional nanoparticles such as Prussian blue, Au nanorods, Pt NPs, *etc.*, (Manikandan et al., 2013; Zeng et al., 2013; Liu et al., 2018). These results suggested that MPDA NPs could serve as good photothermal agents for NIR-I-induced antibacterial activity.

### 3.3 *In Vitro* antibacterial activity assay

**FIGURE 3:**
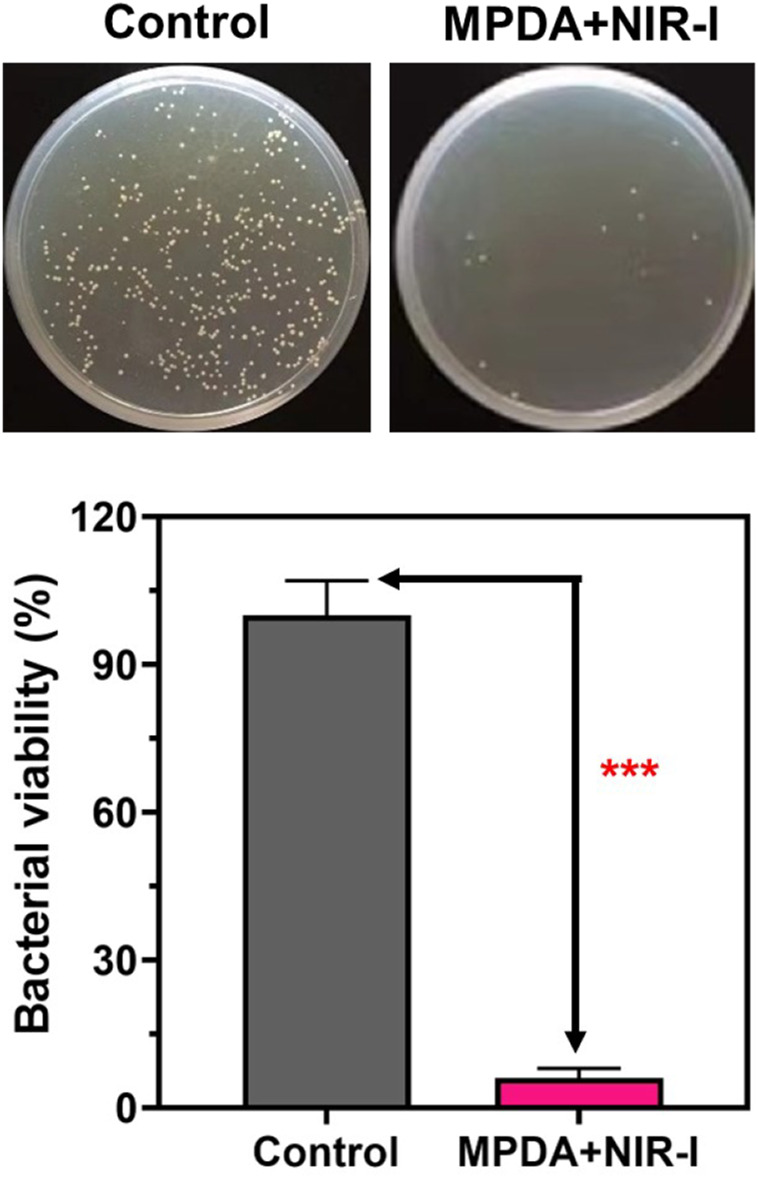
Antibacterial activity of MPDA *in vitro*. Typical agar plate photographs of bacterial colony formed by *S. aureus* after processing by MPDA (200 μg ml^−1^) with and without NIR-I (808 nm, 1 W cm^−2^, 6 min). Corresponding quantitative data of *S. aureus* survival rate after different treatments determined by standard plate counting method. Data are presented as mean ± SD (*n* = 3), ***p < 0.001.

Fascinated by the photothermal results, the *in vitro* antibacterial performance of MPDA NPs against *S. aureus* was evaluated by employing the standard spread plate method. Following the treatment of the *S. aureus* membrane with 200 μg ml^−1^ of MPDA NPs suspension under (808 nm, 1 W cm^−2^,6 min), compared to the control group without NIR-I irradiation, MPDA + NIR-I displayed an enhanced antibacterial efficacy with no effect in the control group Figure 3. PDAs can block bacteria’s nutrient supply and growth-acting barriers and the dopamine benzene ring and some active groups of bacteria to induce toxicity (Waite and Tanzer, 1981; Lee et al., 2006; Ji et al., 2016; Zhao et al., 2022). Though it was obvious that the NIR could equally kill the *S. aureus* the temperature of the NIR-I alone and the time of exposure would not have been enough to cause such an effect. This antibacterial efficiency can therefore be ascribed to the synergistic effect of the MPDA NPs and the NIR-I irradiation. The NIR-I activity could easily impede processes of the bacterial activity which can easily permit the effective action of the MPDA NPs, blocking growth activities and inducing selective toxicity *via* surface active groups.

### 3.4 *In Vivo* antibacterial performance

**FIGURE 4:**
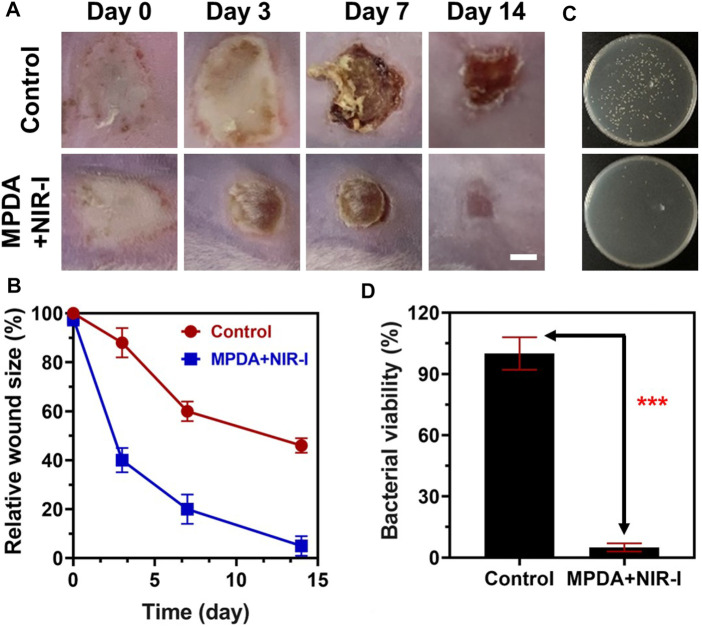
Anti-infective therapy of MPDA *in vivo* with subcutaneous abscess model. **(A)** Representative photographs of the mice with subcutaneous abscesses on the 0, 3^rd^, 7^th^, and 14th day post-treatment. Scale bar, 2 mm. **(B)** Corresponding quantitative analysis of the infected area with various therapies. **(C)** Representative photographs of the bacterial colony from bacteria-infected tissues with various therapies on the 14th day. **(D)** Corresponding quantitative analysis of the number of bacterial colonies from bacteria-infected tissues with various therapies on the 14th day. Data are presented as mean ± SD (*n* = 3), ***p < 0.001.

Upon MPDA NPs achieving excellent antibacterial performance *in vitro*, a mice model of *S. aureus* injected subcutaneous abscess (deep bacterial infection model) was employed to evaluate the effect *in vivo*. 200 μg ml^−1^ of PBS and MPDA NPs suspension were subcutaneously injected into the *S. aureus* abscess, and the treatment group was exposed to (808 nm, 1 W cm^−2^, 6 min). As shown in Figure 4A, from Day 0 to day 7 scars appeared in all groups with the scars becoming dark as time progressed. Following treatment for 14 days, the scar rate of MPDA NPs + NIR-I was significantly reduced and better compared to the control group after Day 14, with the scar of the MPDA NPs + NIR-I group almost completely vanishing Figure 4B. The MPDA NPs + NIR-I combination significantly eradicated *S. aureus* and healed the *S. aureus* infected abscess wound with an insignificant number of bacteria present at the infected sight Figures 4C, D. These results demonstrated that the MDPA NPs under NIR-I could effectively eradicate wound infections and at the same time enhance wound healing.

### 3.5 Histopathological analysis

**FIGURE 5:**
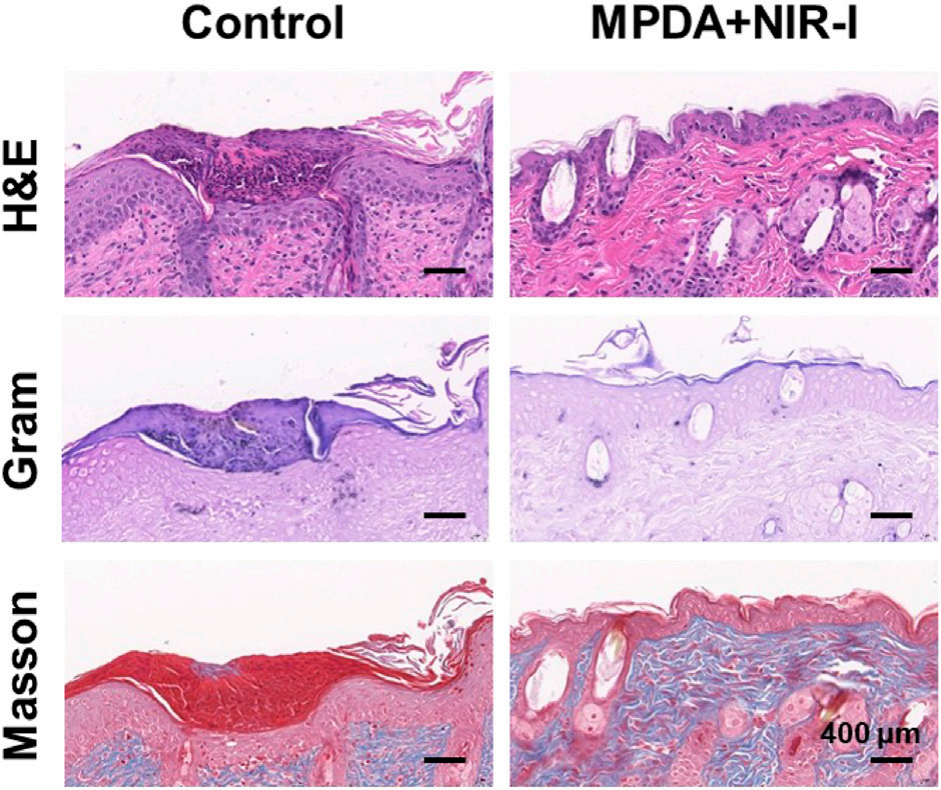
Histological analysis of abscess recovery process. Representative photographs of H&E, Gram, and Masson staining on the 14th day.

**FIGURE 6:**
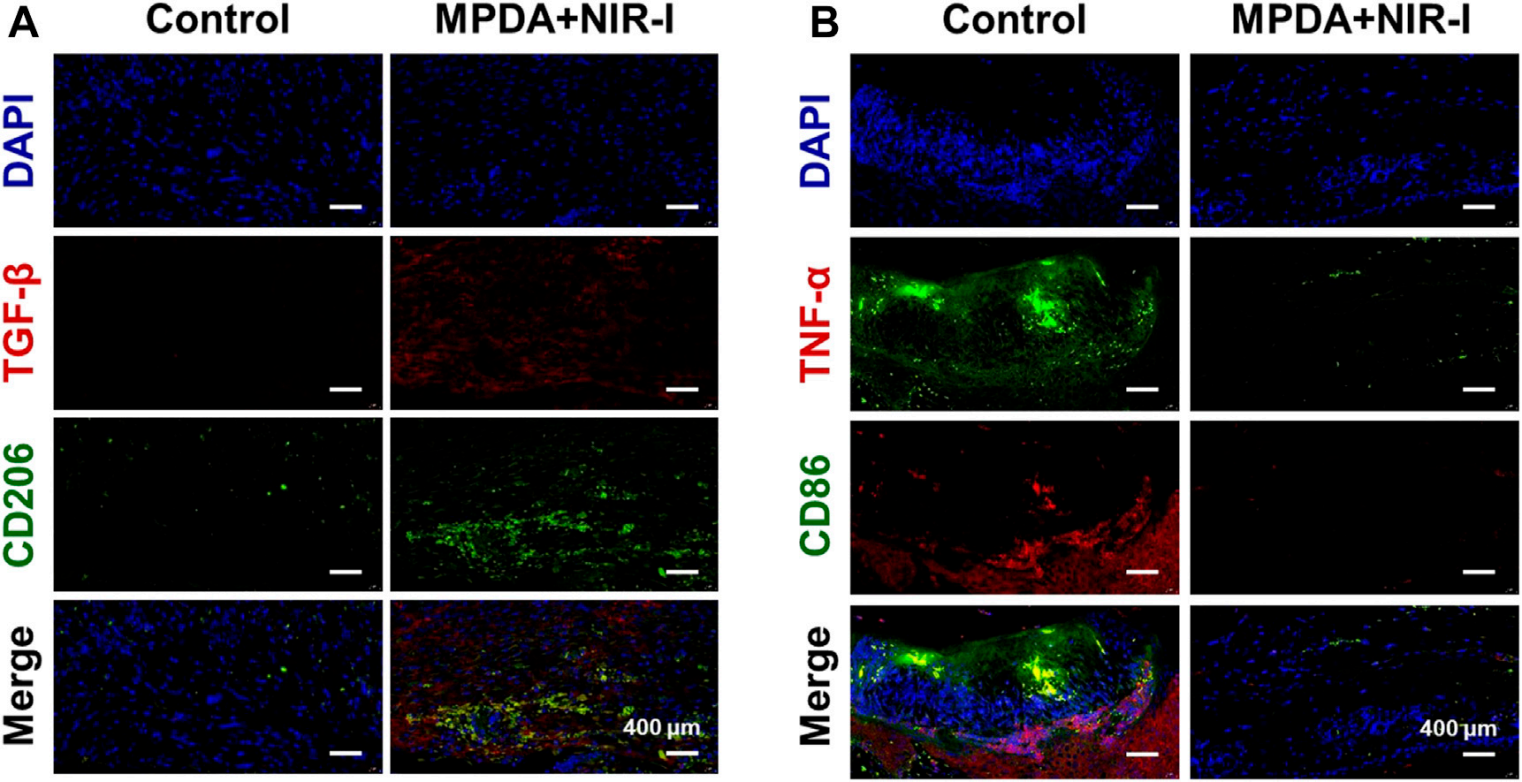
Immunofluorescence analysis of abscess recovery process. Representative photographs of immunofluorescence staining of **(A)** TGF-β/CD206 and **(B)** TNF-α/CD86 on the 14th day.

Further analysis of the synergistic effect of MPDA NPs and NIR-I for the treatment of *S. aureus* infected abscess was assessed using H&E, Gram, and Masson staining. As shown in Figure 5, an abundant neutrophil indicated the accumulation of inflammatory cells as a result of the induced infection. However, MPDA NPs + NIR-I remarkably showed re-epithelization with almost intact epidermal layers which indicated good therapeutic efficacy. Also, almost all *S. aureus* in the MPDA NPs + NIR-I group was completely eradicated based on the Gram staining, Figure 5. Additionally, massive collagen deposition was evident in the MPDA NPs + NIR-I group, an indication of the reconstitution and remodeling of tissues of the skin. The results of Masson staining analysis showed that obvious collagen fibers and a large number of hair follicle structures appeared after treatment, indicating that the skin healing was accelerated. These results displayed the MPDA NPs under photothermal therapy as a good therapeutic agent. Furthermore, as the healing process of an infected would involve stages (anti-infection, anti-inflammation, and tissue regeneration), the inflammation changes of the wound were evaluated after 14 days of treatment. Immunofluorescence was used to analyze the expression of pro-inflammatory and anti-inflammatory cytokines. As shown in Figure 6A *,* TGF-β and CD206 expression in MPDA NPs + NIR demonstrated a bright fluorescence which depicted an enhanced expression. Meanwhile, TNF-α and CD86 expression were significantly reduced, which indicated the remarkable inflammatory reaction Figure 6B. The reason for the above results is mainly attributed to the highly effective antibacterial activity of MPDA NPs, which can quickly remove bacteria from infected tissues through thermal effect, to reduce inflammation and accelerate wound healing. These results confirm that the combination of MPDA NPs + NIR can induce an anti-infection and anti-inflammatory action for the effective healing of a bacteria-infected wound.

### 3.6 Biosafety evaluation of MPDA NPs

**FIGURE 7:**
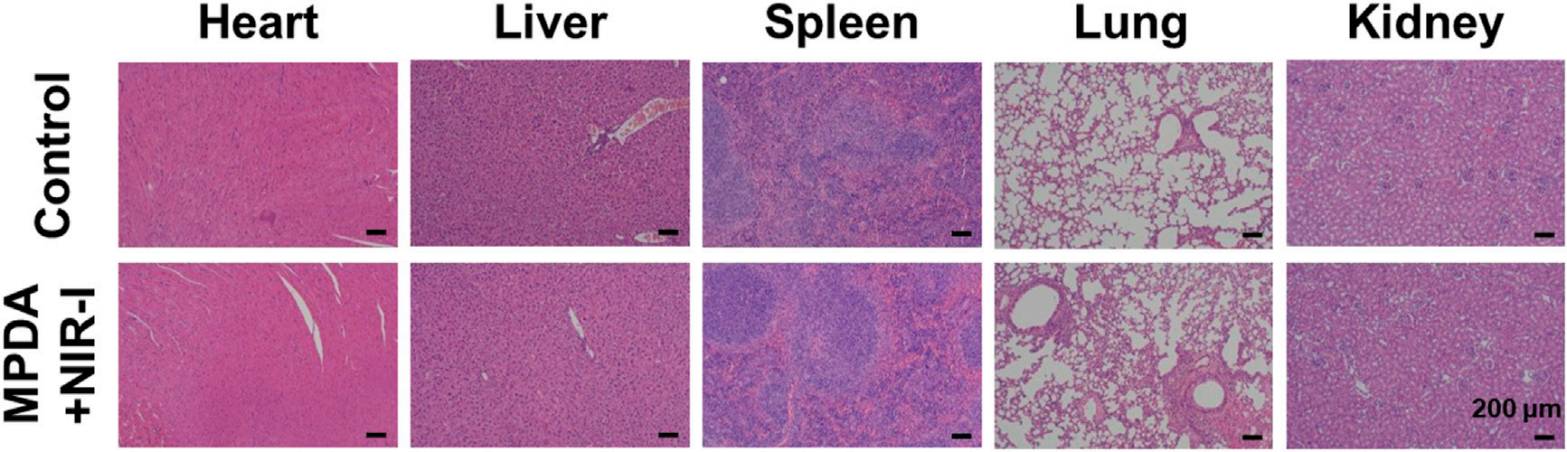
H&E staining images of major organs (heart, liver, spleen, lung, and kidney) of healthy mice after subcutaneous injection of PBS and MPDA + NIR-I on the 14th day after different treatments. Scale bars: 100 μm.

**FIGURE 8:**
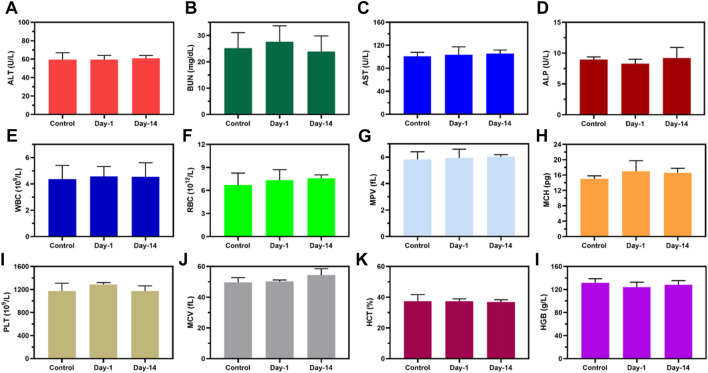
Biosafety evaluation of MPDA. **(A–D)** Blood biochemistry and **(E–L)** blood routine analysis of healthy mice after subcutaneous injection of PBS (Control) and MPDA (day 1 and day 14).

To investigate the *in vivo* biosafety H&E staining was used to confirm the toxicity of MPDA NPs + NIR to the mice, Figure 7. The histological study revealed no obvious pathological abnormalities in the main organs (heart, liver, spleen, lungs, and kidney) proving the biocompatibility of the MPDA NPs even under NIR-I. Also blood chemical indicators: aminotransferase (ALT), blood urea nitrogen (BUN), aspartate aminotransferase (AST), and alkaline phosphatase (ALP) showed no obvious blood toxicity Figures 8A–D. Similarly, blood routine examination: white blood cell (WBC), red blood cell (RBC), mean platelet volume (MPV), mean corpuscular hemoglobin (MCH), platelet count (PLT), mean corpuscular volume (MCV), hematocrit (HCT) and hemoglobin (HGB) exhibited no significant abnormality Figure 8E–L. These results proved the negligible toxicity induced by the MPDA NPs under NIR-I, demonstrating good biocompatibility.

## 4 Conclusion

In summary, MPDA NPs were fabricated *via* emulsion-induced interface polymerization. The activity of MPDA NPs was efficiently enhanced under NIR-I by this property the MPDA *via* effective thermal-killing eradicated the *S. aureus in vitro*. Notably, this synergistic therapeutic effect was proven in *S. aureus*-infected abscesses *in vivo*, eliminating bacterial infections with subsequent wound healing. MPDA NPs’ intriguing property which spans from good biocompatibility to good photothermal performance present it as a new platform promising for antibacterial activity, wound healing, and future biomedical application.

## Data Availability

The original contributions presented in the study are included in the article/Supplementary Material, further inquiries can be directed to the corresponding author.
